# Visualising and modelling changes in categorical variables in longitudinal studies

**DOI:** 10.1186/1471-2288-14-32

**Published:** 2014-02-27

**Authors:** Mark Jones, Richard Hockey, Gita D Mishra, Annette Dobson

**Affiliations:** 1 Centre for Longitudinal and Life Course Research, School of Population Health, University of Queensland, Brisbane, Australia; 2 Public Health Building, Herston Road Herston, Brisbane, Qld, 4006, Australia

**Keywords:** Categorical variables, Graphical methods, Longitudinal studies, Marginal distribution, Nominal regression, Transition probabilities

## Abstract

**Background:**

Graphical techniques can provide visually compelling insights into complex data patterns. In this paper we present a type of *lasagne* plot showing changes in categorical variables for participants measured at regular intervals over time and propose statistical models to estimate distributions of marginal and transitional probabilities.

**Methods:**

The plot uses stacked bars to show the distribution of categorical variables at each time interval, with different colours to depict different categories and changes in colours showing trajectories of participants over time. The models are based on nominal logistic regression which is appropriate for both ordinal and nominal categorical variables. To illustrate the plots and models we analyse data on smoking status, body mass index (BMI) and physical activity level from a longitudinal study on women’s health. To estimate marginal distributions we fit survey wave as an explanatory variable whereas for transitional distributions we fit status of participants (e.g. smoking status) at previous surveys.

**Results:**

For the illustrative data the marginal models showed BMI increasing, physical activity decreasing and smoking decreasing linearly over time at the population level. The plots and transition models showed smoking status to be highly predictable for individuals whereas BMI was only moderately predictable and physical activity was virtually unpredictable. Most of the predictive power was obtained from participant status at the previous survey. Predicted probabilities from the models mostly agreed with observed probabilities indicating adequate goodness-of-fit.

**Conclusions:**

The proposed form of *lasagne* plot provides a simple visual aid to show transitions in categorical variables over time in longitudinal studies. The suggested models complement the plot and allow formal testing and estimation of marginal and transitional distributions. These simple tools can provide valuable insights into categorical data on individuals measured at regular intervals over time.

## Background

With the increasing interest in longitudinal and life-course studies, it is desirable to develop graphical techniques for visualising and exploring complex patterns within groups of participants over the course of a study. However graphical presentation of variables measured at different times in longitudinal studies can be challenging. To be useful a graphical technique should be simple to implement and interpret, provide valuable insights into the structure of the data, and be viable for large sample sizes.

A well-known method for graphically displaying longitudinal data is the *spaghetti* plot [1] where individual subject’s measurements of a repeated outcome are shown chronologically over time. This graphical method is simple and effective at showing changes in a variable for individuals. However it is only appropriate for continuous data and small sample sizes. Plotting a large number of trajectories can lead to multiple intersecting lines that fail to show important patterns in the data.

Recently the *lasagne* plot has been developed that is claimed to address the limitations of the *spaghetti* plot [2]. Based on heat maps [3] each subject’s trajectory over time is shown in a horizontal layer with colour used to depict the magnitude of the response value at each time-point. Data for groups of individuals are then stacked on top of each other in layers, hence the term, *lasagne*.

In this paper we describe a form of lasagne plot for showing changes in categorical variables for participants in longitudinal studies. In addition to the plot, we recommend including a table showing marginal distributions over time. To complement the plot we illustrate the use of standard statistical models that estimate marginal and transitional distributions of categorical variables over time consistent with the patterns depicted graphically or in the table.

## Methods

### Example data

To illustrate the construction and interpretation of the plots, data from the Australian Longitudinal Study on Women’s Health (ALSWH) [4] were used. This ongoing survey of 40,000 adult women in three age groups was initiated in 1996 and has five or more waves of data for each of the three age group cohorts. The study has been approved by Ethics Committees at the University of Queensland and University of Newcastle. We used data from the women born between 1973 and 1978 to illustrate our proposed methods.

Self-reported data on smoking status, body mass index (BMI) and physical activity level were obtained from participants in 1996, 2000, 2003, 2006 and 2009. Smoking status is categorised as never smoker, current smoker, or ex-smoker; BMI is categorised as healthy or underweight (BMI ≤ 25.0), overweight (25.0 < BMI ≤ 30.0), or obese (BMI > 30.0); and physical activity is categorised as low/sedentary (inactive), moderate activity or high activity [5]. As the proportion of participants classified as underweight (BMI < 18.5) was very small and diminished over time, for simplicity we combined this category with the healthy weight category (18.5 < BMI ≤ 25.0) and refer to the combined category as just healthy weight for the remainder of the manuscript. To simplify the illustration we have restricted analysis to those participants with complete data for each of the categorical variables. For smoking status there was a constraint that current or ex-smokers could not be categorised as never smokers at a later survey. Comparable data for physical activity were not available for the first survey hence we restrict our analyses to data from surveys 2 to 5.

### The plot

The proposed plot uses stacked bars to show the distribution of categorical variables across surveys, with different colours to depict different categories and changes in colours over waves depicting trajectories of groups of participants over time. The plot shows transitional distributions of categorical variables across surveys hence the status of participants can be tracked over the course of the study. As well as longitudinal changes represented by the stacked bars, cross sectional data can also be presented in tabular form above each bar. The plot and table can be produced using standard software such as SAS Statistical Graphics (SAS Institute Inc., Cary, NC).

### Statistical models

To estimate the marginal and transitional probabilities for categorical variables we used nominal logistic regression models [6]. These models include binary or binomial logistic regression for variables with just two categories, as well as models for more than two categories. For ordinal categorical variables assumptions such as proportional odds are needed to make use of the additional information about the natural order of the categories.

As the data are longitudinal it is necessary to take into account the correlation between successive measurements on the same individuals. This can be done using mixed models for individuals. Until recently however such models for categorical outcomes could not be readily fitted with standard software. An alternative approach is to model the data as independent observations but use variance estimates robust to this assumption.

For this paper, to complement the proposed plot, nominal logistic regression models were fitted using Stata/IC, version 12.0 for Windows (StataCorp, College Station, TX) with robust variances. With Stata version 13 mixed models could have been fitted.

For marginal models the general formulation is:

(1) $$\mathrm{logit}\;\pi_{j}=\log\;\left(\frac{\pi_{j}}{\pi_{1}}\right)=x_{j}^{T}\beta_{j}$$

where *j = 2, …, J* categories; *π*_*j*_ is the probability of being in category *j*; *π*_*1*_ is the probability of being in the reference category; *x*_*j*_^*T*^ is the transpose of the matrix of predictor variables for each participant; and *β*_*j*_ is the vector of coefficients to be estimated for each category *j*.

Models to estimate marginal probabilities included variables for survey wave. Goodness-of-fit of the models was assessed by comparing estimated and observed marginal probabilities. Ordinal models could have potentially been fitted for the ordinal outcomes BMI group and physical activity level however to facilitate comparison across the three outcomes of interest we chose to fit nominal models for all three outcome variables.

Models to estimate transition probabilities included predictor variables that indicated outcome status at previous surveys [7]. Goodness-of-fit was assessed by comparing estimated and observed transition probabilities as well as calculating McFadden’s pseudo R^2^ which is an estimate of the magnitude of improvement of the fitted model compared to the uninformative or null model [8]. We also calculated the proportion of correct predictions provided by the final model and contrasted the result with the proportion of correct predictions from an uninformative model where no explanatory variables were fitted. The delta method was used to estimate standard errors for transition probabilities so that 95% confidence intervals could be calculated.

To guide our decision on how many previous surveys to include as explanatory information in the models, we estimated variance inflation factors (VIF) and percentage increases in log likelihood. We preferred percentage increases in log likelihood to the more common approach of using absolute increases to assess model fit because they are more informative in terms of predictability for individuals. As an additional visual tool to illustrate distributions of outcome variables over time, probability tree diagrams depicting proportions of participants in each response category at each wave were used to assist with constructing the transition models.

## Results

### The plot

**Figure 1:**
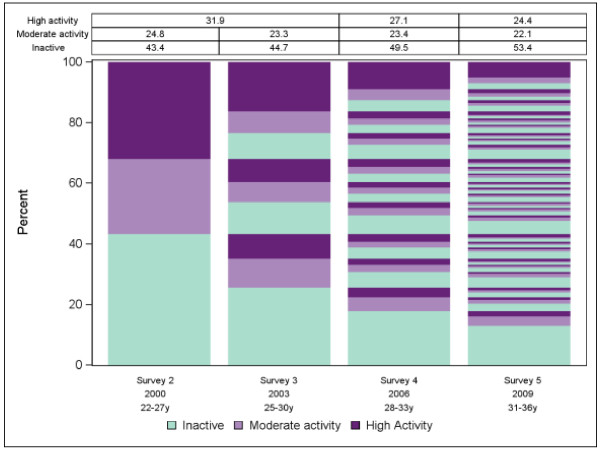
Plot and marginal distribution table of physical activity level over survey wave for the Australian Longitudinal Survey of Women’s Health.

**Figure 2:**
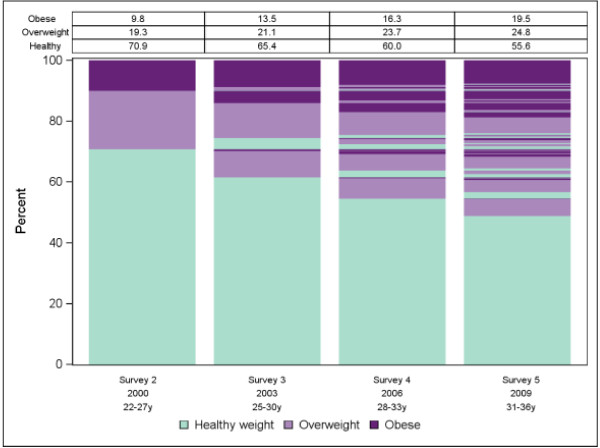
Plot and marginal distribution table of body mass index group over survey wave for the Australian Longitudinal Survey of Women’s Health.

**Figure 3:**
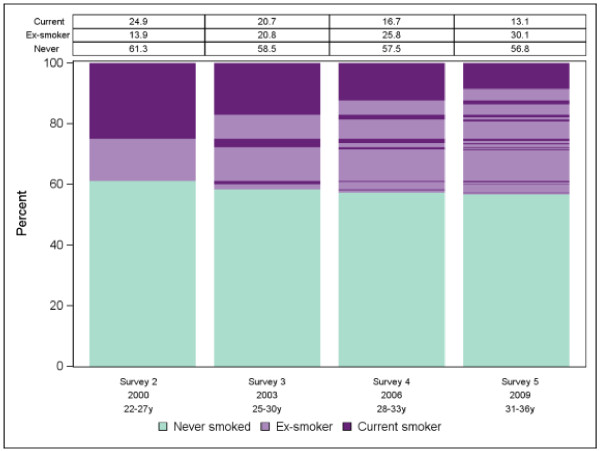
Plot and marginal distribution table of smoking status over survey wave for the Australian Longitudinal Survey of Women’s Health.

The proposed plots are shown in Figures 1, 2 and 3 for physical activity level, BMI group and smoking status respectively. Informally comparing the three outcome variables, it appears participants were more likely to change physical activity level between surveys than BMI group or smoking status. However BMI category cannot change as quickly as levels of physical activity or smoking status. Also it was not possible to become a never smoker after being a smoker. The plots suggest predictability of physical activity level for individuals over time would be low whereas predictability would be better for BMI group and perhaps even better for smoking status. See Additional file 1 for SAS code we used for the smoking status plot. To investigate predictability of individuals over time further we used more formal procedures.

### Marginal models

Marginal nominal logistic models for all three outcome variables showed approximately linear changes in log relative risk ratios (RRR) over surveys hence survey was fitted as a numerical variable:

(2) $$\mathrm{logit}\;\pi_{j}=\beta_{1j}+\mathrm{Survey}\times \beta_{2j}$$

where *Survey = 2, …, 5*.

Predicted probabilities from the fitted models had consistently high agreement with the observed probabilities with absolute differences within 1-2% in all cases (data not shown). Compared to being inactive the relative risk of being in the moderate physical activity category decreased at each survey by 7% (RRR = 0.93; 95% CI: 0.90, 0.95) and the relative risk of being in the high physical activity category decreased at each survey by 14% (RRR = 0.86; 95% CI: 0.85, 0.88). With healthy weight as the reference group, the relative risk of being in a higher BMI category increased at each survey (RRR = 1.17; 95% CI: 1.15, 1.20 for overweight and RRR = 1.32; 95% CI: 1.29, 1.34 for obese). For smoking status, where never smokers was the reference category, the relative risk of being an ex-smoker increased (RRR 1.17; 95% CI: 1.15, 1.19) whereas the relative risk of being a current smoker decreased at each survey (RRR 0.81; 95% CI: 0.79, 0.82).

### Transitional models

Results for physical activity showed VIFs that were less than 1.5 for inclusion of explanatory variables at any number of previous surveys. Percentage increases in log likelihood were modest generally and less than 1% for two and three previous surveys (Table 1). Based on these results we included just the previous survey in the transition model for physical activity. Our proposed transition model equation for physical activity was:

(3) $$\begin{array}{l} \mathrm{logit}\;\pi_{j}=\beta_{1j}+I{\left(\mathrm{Moderate}\right)}_{-1}\times \beta_{2j}\\ \;+I{\left(\mathrm{High}\right)}_{-1}\times \beta_{3j} \end{array}$$

where *I(Moderate)*_-1_ indicates moderate activity level at the previous survey and *Ι*(*High)*_-1_ indicates high activity level at the previous survey.

**Table 1:** Percentage changes in log likelihood

**Variable at survey wave 5**	**Null model**	**Previous survey**	**Two surveys previous**	**Three surveys previous**
Physical activity level	-8062.1	-7703.1	-7648.9	-7601.2
		4.5%	+0.6%	+0.6%
BMI group	-8644.0	-4811.2	-4562.8	-4506.7
		44.3%	+2.9%	+0.6%
Smoking status	-10906.3	-2588.4	-2449.6	-2424.8
		76.3%	+1.2%	+0.2%

Model estimates showed that previous moderate activity was associated with an 80% increased relative risk of current moderate activity (RRR 1.84; 95% CI: 1.65, 2.04) and more than a doubling in relative risk of current high activity (RRR 2.15; 95% CI: 1.93, 2.40). In addition previous high activity is associated with a more than doubling in relative risk of current moderate activity (RRR 2.33; 95% CI: 2.09, 2.59) and a more than five-fold increase in relative risk of current high activity (RRR 5.40; 95% CI: 4.87, 5.99). Pseudo R^2^ for the fitted model was 4.5% and the proportion of correct predictions was 56% (compared to 53% correct predictions for an uninformative model) indicating a poor predictability of physical activity level for individuals based on previous survey results. However predicted probabilities from the fitted model agreed with the observed probabilities to within 1% for all comparisons indicating a good overall model fit. Estimated transition probabilities showed that previous moderate activity was associated with a 47% (95% CI: 45%, 48%) probability of current low or sedentary activity, a 27% (95% CI: 26%, 29%) probability of current moderate activity and a 26% (95% CI: 24%, 28%) probability of current high activity. Previous high activity was associated with a 32% (95% CI: 30%, 33%) probability of current low or sedentary activity, a 24% (95% CI: 22%, 25%) probability of current moderate activity and a 44% (95% CI: 43%, 46%) probability of current high activity. Previous low or sedentary activity was associated with a 63% (95% CI: 62%, 64%) probability of current low or sedentary activity, a 20% (95% CI: 19%, 21%) probability of current moderate activity and a 16% (95% CI: 15%, 17%) probability of current high activity.

For BMI group, based on all VIFs being less than 3 and percentage increases in log likelihood as shown in Table 1, the BMI categories for the two previous surveys were included in the transition model. The reference category was chosen to be the overweight group as this ensured the estimated relative risk ratios and standard errors were stable. The transition model equation for BMI group was:

(4) $$\begin{array}{l} \mathrm{logit}\;\pi_{j}=\beta_{1j}+I{\left(\mathrm{Healthy}\right)}_{-1}\times \beta_{2j}+I{\left(\mathrm{Obese}\right)}_{-1}\times \beta_{3j}\\ \;+I{\left(\mathrm{Healthy}\right)}_{-2}\times \beta_{4j}+I{\left(\mathrm{Obese}\right)}_{-2}\times \beta_{5j} \end{array}$$

where *Ι (Healthy)*_*-1*_ indicates healthy weight at the previous survey, *Ι (Obese)*_*-1*_ indicates obesity at the previous survey, *Ι (Healthy)*_*-2*_ indicates healthy weight two surveys previously, and *Ι (Obese)*_*-2*_ indicates obesity two surveys previously.

Table 2 shows estimated relative risk ratios and 95% confidence intervals obtained from the transition model. Pseudo R^2^ for the model was 0.47 with 81% correct predictions (compared to 59% correct predictions for an uninformative model) indicating moderate predictability of current BMI group for individuals based on BMI group at the two previous surveys. Predicted and observed transitional probabilities showed only moderate agreement, although some of these categories included low numbers of participants (Additional file 2: Figure S1).

**Table 2:** Relative risk ratios based on transitional model for BMI group (reference = overweight)

**Outcome**	**Predictor variable**	**Relative risk ratio (95% confidence interval)**
Healthy weight	Previously healthy weight	14.2 (12.2, 16.5)
Healthy weight	Previously obese	0.83 (0.47, 1.44)
Healthy weight	Healthy weight two surveys previously	5.11 (4.22, 6.18)
Healthy weight	Obese two surveys previously	0.68 (0.35, 1.35)
Obese	Previously healthy weight	0.20 (0.13, 0.29)
Obese	Previously obese	11.6 (9.20, 14.5)
Obese	Healthy weight two surveys previously	0.59 (0.47, 0.75)
Obese	Obese two surveys previously	2.92 (2.25, 3.80)

**Figure 4:**
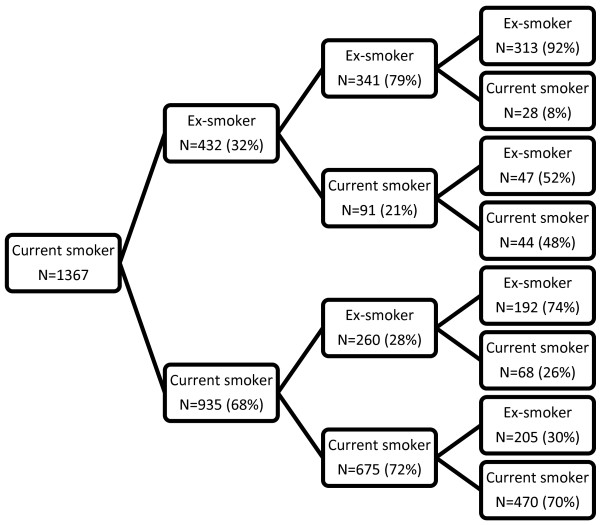
Probability tree for current smoker over survey waves 2–5.

For smoking status, VIFs of between 6 and 24 indicated strong multicollinearity when more than one previous survey was included as explanatory information in a transition model. However we were able to include an explanatory variable indicating whether or not a participant was an ex-smoker two surveys previously as this did not result in multicollinearity and added useful predictive information to the model. Figure 4 shows a probability tree diagram for current smokers illustrating the additional predictive value of including being an ex-smoker two surveys previously as a predictor variable. In contrast, being a current smoker two surveys previously added little additional predictive value. Transitions from being an ex- or current smoker to never having smoked are not possible hence current smoker was chosen as the reference category and predictor coefficients indicating previous smoking status of never smokers were constrained to be (structurally) zero. Some participants reported never smoking after being classified as an ex-smoker or current smoker at earlier surveys. These participants were reclassified as ex-smokers. The transition model (shown below) included indicator variables for whether the participant was a current smoker in the previous survey, an ex-smoker at the previous survey, or an ex-smoker at the previous two surveys.

(5) $$\begin{array}{l} \mathrm{logit}\;\pi_{j}=\beta_{1j}+I{\left(\mathrm{Ex}\right)}_{-1}\times \beta_{2j}+I{\left(\mathrm{Current}\right)}_{-1}\times \beta_{3j}\\ \;+I{\left(\mathrm{Ex}\right)}_{-1,-2}\times \beta_{4j} \end{array}$$

where _-1_ indicates status at the previous survey, _-1,-2_ indicates ex-smoker for both previous surveys.

Estimates obtained from the model showed that being an ex-smoker at the previous survey (but not being an ex-smoker two surveys previously) was associated with a doubling in relative risk of being an ex-smoker currently (RRR 2.01; 95% CI: 1.12, 3.63) whereas being an ex-smoker for both previous surveys was associated with a 12-fold increased relative risk of being an ex-smoker currently (RRR 12.8; 95% CI: 6.95, 23.5). Being a current smoker at the previous survey was associated with a 72% lower relative risk of being an ex-smoker currently (RRR 0.28; 95% CI: 0.16, 0.50). The final model had pseudo R^2^ = 0.77 with 91% correct predictions (compared to 52% correct predictions for an uninformative model) hence smoking status for the previous surveys was highly predictive of current status for individuals. Predicted and observed transitional probabilities agreed to within 1% in all cases hence goodness-of-fit statistics indicated the model fitted the observed data well. Predicted probabilities showed previous never smokers had 99% (95% CI: 98.8%, 99.3%) chance of being a never smoker at the current survey, 0.6% (95% CI: 0.4%, 0.8%) chance of being an ex-smoker and 0.3% (95% CI: 0.1%, 0.5%) chance of being a current smoker. Previous current smokers had 34% (95% CI: 32%, 37%) chance of being an ex-smoker in the current survey and 66% (95% CI: 63%, 68%) of being a current smoker. An ex-smoker for the two previous surveys had 4% (95% CI: 3%, 5%) chance of being a current smoker at the current survey and 96% (95% CI: 95%, 97%) chance of being an ex-smoker. However for ex-smokers at the previous survey, who were not ex-smokers two surveys previously, the transition probabilities were 21% (95% CI: 18%, 24%) for current smoking and 79% (95% CI: 76%, 82%) for ex-smoking.

## Discussion

The plot we have illustrated visually depicts changes in categorical variables for individuals over time. However the marginal distribution at follow up time-points is not as clearly shown, therefore we recommend inclusion of a table above the stacked bars showing the marginal distribution at each time-point. Simple nominal logistic regression models can be used to formalise the visual information provided by the plot and estimate marginal and transitional probabilities as well as relative effects. Probability tree diagrams are useful in helping develop the models.

Transitional probabilities, in particular, provide useful and easily interpretable summary information. For example, based on ALSWH data, conditional on being overweight for two previous surveys, Australian women in their twenties had 23% (95% CI: 21%, 26%) probability of being obese at the next survey but only 7% (95% CI: 6%, 9%) probability of being of healthy weight (or underweight). We suggest McFadden’s pseudo R^2^ as a summary measure for assessing predictability of categorical outcomes and, to guide decisions on how many previous measures should be included in the transition models, we propose using variance inflation factors and percentage increases in log likelihood.

*Spaghetti* plots are very useful for showing changes in a numerical variable for a limited number of individuals over time but are not applicable for categorical data or large numbers of individuals. *Lasagne* plots have been proposed as an alternative to *spaghetti* plots for categorical data and/or many individuals. There are however several other methods for graphically representing categorical data but they have a number of limitations. For example, in the mosaic plot the relative frequency of each level of a variable and its relationship to another variable is represented by a mosaic of tiles [9]; see Additional file 3: Figure S2. A variable degree of shading for each tile is then incorporated to represent the degree of deviation from a null hypothesis of independence. However adding more variables increases complexity and showing the distribution of a categorical variable over multiple waves of a longitudinal survey is not feasible.

Another technique is known as parallel sets [10]; a similar concept to Sankey diagrams [11]. In these diagrams the relationship between variables is shown using parallelograms whose width is proportional to the frequencies involved (Additional file 4: Figure S3). Parallel sets are appropriate for categorical data collected on large numbers of participants over multiple surveys but the plot lacks simplicity and software to produce the figures is not readily available.

The *lasagne* plot we illustrate offers some advantages over these alternative methods in terms of ease of depiction and interpretation. But, irrespective of which graphical method is used, the information obtained is only descriptive hence the need for methods that allow formal testing and estimation.

To simplify our illustration we restricted analysis to individuals who provided complete responses over four surveys. However this restriction could be relaxed to include all participants. In this case missing data could form an additional category and be included in the plot, tabulation and models. The addition of a missing data category could provide additional insights into the data. For example, it could show that certain categories in previous surveys are associated with increased risk of missing data in subsequent surveys. It may also be of interest to tabulate the patterns of missing data across surveys. We illustrate the inclusion of missing data as a category in the graphical analysis of BMI categorised into healthy, overweight/obese, or missing (Additional file 5: Figure S4). The plot suggests previous missingness predicts current missingness but missingness does not appear to be associated with the other BMI categories of healthy weight and overweight/obese.

A limitation of the lasagne plot is that it is not feasible to include a large number of categories. In our example we used three categories. Including more categories would make interpretation increasingly difficult. We therefore recommend four categories at most. If there are more than four categories then we recommend collapsing the data into fewer categories. For physical activity level, for example, we collapsed sedentary and low activity into “inactive”. Continuous variables could also be summarised using these methods however they would require categorisation with consequential loss of detail. A further limitation is that the number of categories shown at later time-points can be high making interpretation difficult. If this is the case we suggest making separate plots of transitions from each individual category at the initial survey to supplement the overall plot. We illustrate this idea for smoking status in Additional file 6: Figures S5 and Additional file 7: S6. Finally we have illustrated the plot and models with data from a longitudinal survey where the participants have been assessed at regular periods over time. If data had been collected at irregular time-points, our methodology may not be appropriate. Despite these limitations, we believe the plot and models are appropriate in general for categorical variables collected in longitudinal studies.

## Conclusions

The lasagne plot we illustrate provides a simple way to show transitions in the status of individuals observed longitudinally. The regression models we suggest complement the plots and allow formal testing and reporting of marginal and transitional distributions. These analytical tools can be implemented in standard statistical software such as SAS and Stata and can provide valuable insights into categorical variables measured on individuals at regular intervals over time.

## Abbreviations

ALSWH: Australian Longitudinal Study of Women’s Health; BMI: Body mass index; VIF: Variance inflation factor; RRR: Relative risk ratio.

## Competing interest

The authors declare that they have no competing interest.

## Authors’ contribution

RH developed the plot methodology; MJ developed the modelling strategy and wrote the manuscript; GM and AD oversaw the project, provided supervision as well as critical input into the design and implementation; all authors reviewed and/or revised the manuscript and have approved it for submission.

## Pre-publication history

The pre-publication history for this paper can be accessed here:

http://www.biomedcentral.com/1471-2288/14/32/prepub

## Supplementary Material

Additional file 1 SAS code for generating smoking status plot. Click here for file

Additional file 2: Figure S1 Probability tree diagram for BMI group with observed and estimated transitional probabilities and 95% confidence intervals in brackets. Click here for file

Additional file 3: Figure S2 Mosaic plot of smoking status at survey wave 1 compared to wave 2. Click here for file

Additional file 4: Figure S3 Parallel sets diagram of smoking status transitions from survey waves 1 to 5. Click here for file

Additional file 5: Figure S4 Plot and marginal distribution table of body mass index group with a missing category over survey wave for the Australian Longitudinal Survey of Women’s Health. Click here for file

Additional file 6: Figure S5 Plot and marginal distribution table of smoking status over survey wave for ex-smokers at survey wave 2. Click here for file

Additional file 7: Figure S6 Plot and marginal distribution table of smoking status over survey wave for current smokers at survey wave 2. Click here for file
