# Supplementary material for: Visualising and modelling changes in categorical variables in longitudinal studies
Source: BMC Med Res Methodol. 2014 Feb 27;14:32. doi: 10.1186/1471-2288-14-32 (PMC3938907; doi:10.1186/1471-2288-14-32)
Supplement: Additional file 1 — SAS code for generating smoking status plot. [file 1471-2288-14-32-S1.docx]

Appendix: SAS code for generating smoking status plot

data yngsmk;

set yngdata;/*individual survey data containing smoking status at each survey */

/* smoking status at each survey smk1-smk5 coded 0=never smoked, 1=ex smoker, 2=current smoker */

/* create smoking pattern strings at each survey*/

smkstr5=put(smk1,1.)||put(smk2,1.)||put(smk3,1.)||put(smk4,1.)||put(smk5,1.);

smkstr4=substr(smkstr5,1,4);

smkstr3=substr(smkstr5,1,3);

smkstr2=substr(smkstr5,1,2);

smkstr1=substr(smkstr5,1,1);

drop i;

run;

/* output frequencies of smoking patterns to datasets */

proc freq data=yngsmk;

where smk1^=. and smk2^=. and smk3^=. and smk4^=. and smk5^=. ;

table smkstr1/out=outyng1;

table smkstr2/out=outyng2;

table smkstr3/out=outyng3;

table smkstr4/out=outyng4;

table smkstr5/out=outyng5;

run;

/*combine datasets */

data allsmk;

length smkstr $ 5;

set outyng1(in=in1) outyng2(in=in2) outyng3(in=in3) outyng4(in=in4) outyng5(in=in5) ;

if in1 then do;

survey='S1';

smkstr=smkstr1;

end;

else if in2 then do;

survey='S2';

smkstr=smkstr2;

end;

else if in3 then do;

survey='S3';

smkstr=smkstr3;

end;

else if in4 then do;

survey='S4';

smkstr=smkstr4;

end;

else if in5 then do;

survey='S5';

smkstr=smkstr5;

end;

/*indexn controls the colours */

indexn=input(substr(smkstr,length(smkstr)),1.)+1;

label percent='Percent';

run;

/* output smoking status at each survey to datasets */

proc freq data=yngsmk;

where smk1^=. and smk2^=. and smk3^=. and smk4^=. and smk5^=. ;

table smk1/out=smk1;

table smk2/out=smk2;

table smk3/out=smk3;

table smk4/out=smk4;

table smk5/out=smk5;

run;

/*combine datasets */

data allsmk2;

set smk1(in=in1 ) smk2(in=in2) smk3(in=in3) smk4(in=in4 ) smk5(in=in5 ) ;

if in1 then do;

survey='S1';

smkstat=smk1;

end;

else if in2 then do;;

survey='S2';

smkstat=smk2;

end;

else if in3 then do;

survey='S3';

smkstat=smk3;

end;

else if in4 then do;

survey='S4';

smkstat=smk4;

end;

else if in5 then do;

survey='S5';

smkstat=smk5;

end;

percent2=round(percent,0.1);

format percent2 4.1;

drop percent;

run;

proc sort data=allsmk2;

by survey descending smkstat;

run;

proc sort data=allsmk;

by survey;

run;

data allsmk;

merge allsmk allsmk2;

by survey;

run;

/* create axis labels */

Data axislab;

survey= 'S1';

alabel='Survey'; atext='Survey 1'; output;

alabel='Year' ; atext='1996'; output;

alabel='Age'; atext='18-23y'; output;

survey= 'S2';

alabel='Survey'; atext='Survey 2'; output;

alabel='Year' ; atext='2000'; output;

alabel='Age'; atext='22-27y'; output;

survey= 'S3';

alabel='Survey'; atext='Survey 3'; output;

alabel='Year' ; atext='2003'; output;

alabel='Age'; atext='25-30y'; output;

survey= 'S4';

alabel='Survey'; atext='Survey 4'; output;

alabel='Year' ; atext='2006'; output;

alabel='Age'; atext='28-33y'; output;

survey= 'S5';

alabel='Survey'; atext='Survey 5'; output;

alabel='Year' ; atext='2009'; output;

alabel='Age'; atext='31-36y'; output;

label survey='Survey, Year and Age';

run;

proc sort;

by survey;

run;

data allsmk;

merge allsmk axislab;

by survey;

run;

/*define colours */

proc template;

define style newchart;

parent=styles.journal;

style GraphData1 from GraphData1 /

color=cxaaddcc ; /*green*/

style GraphData2 from GraphData2 /

color=cxaa88bb ; /*light purple */

style GraphData3 from GraphData3 /

color=cx662277 ; /*purple */

end;

run;

/*create chart definition using GTL*/

proc template;

define statgraph blockplot2;

begingraph;

/* entrytitle "1973-78 Cohort - Smoking";*/

legendItem type=fill name="ns_marker" /

fillattrs=(color=cxaaddcc )

label="Never smoked" ;

legendItem type=fill name="ex_marker" /

fillattrs=(color=cxaa88bb )

label="Ex-smoker" ;

legendItem type=fill name="cs_marker" /

fillattrs=(color=cx662277 )

label="Current smoker" ;

layout lattice / columns=1 rowweights=(.10 .75 .10 .05) columndatarange=union;

COLUMNAXES;

columnaxis / display=none ;

endcolumnaxes;

blockplot x=survey block=percent2 / class=smkstat

includemissingclass=false

display=(values label outline) valuehalign=center

labelattrs=GraphDataText valueattrs=GraphDataText;

barchart x=survey y=percent / group=smkstr orient=vertical display=(fill) index=indexn;

blockplot x=survey block=atext / class=alabel

includemissingclass=false

display=(values) valuehalign=center

labelattrs=GraphDataText valueattrs=GraphDataText;

discretelegend "ns_marker" "ex_marker" "cs_marker" / border=false;

endlayout;

endgraph;

end;

run;

/* format for smoking status */

proc format;

value smk

0='Never'

1='Ex-smoker'

2='Current';

title ;

/* create graph */

ods listing close;

ods rtf file='yng_smk_new.rtf' style=newchart;

proc sgrender data=allsmk template=blockplot2 ;

format smkstat smk. ;

run;

ods rtf close;

ods listing;
