# Supplementary material for: Visualising and modelling changes in categorical variables in longitudinal studies
Source: BMC Med Res Methodol. 2014 Feb 27;14:32. doi: 10.1186/1471-2288-14-32 (PMC3938907; doi:10.1186/1471-2288-14-32)
Supplement: Additional file 2: Figure S1 — Probability tree diagram for BMI group with observed and estimated transitional probabilities and 95% confidence intervals in brackets. [file 1471-2288-14-32-S2.docx]

Figure S1: Probability tree diagram for BMI group with observed and estimated transitional probabilities and 95% confidence intervals in brackets
