# Supplementary material for: Visualising and modelling changes in categorical variables in longitudinal studies
Source: BMC Med Res Methodol. 2014 Feb 27;14:32. doi: 10.1186/1471-2288-14-32 (PMC3938907; doi:10.1186/1471-2288-14-32)
Supplement: Additional file 3: Figure S2 — Mosaic plot of smoking status at survey wave 1 compared to wave 2. [file 1471-2288-14-32-S3.docx]

Figure S2: Mosaic plot of smoking status at survey wave 1 compared to wave 2
