# Supplementary figures and images for: Visualising and modelling changes in categorical variables in longitudinal studies
Source: BMC Med Res Methodol. 2014 Feb 27;14:32. doi: 10.1186/1471-2288-14-32 (PMC3938907; doi:10.1186/1471-2288-14-32)

Figure S3: Parallel sets diagram of smoking status transitions from survey waves 1 to 5


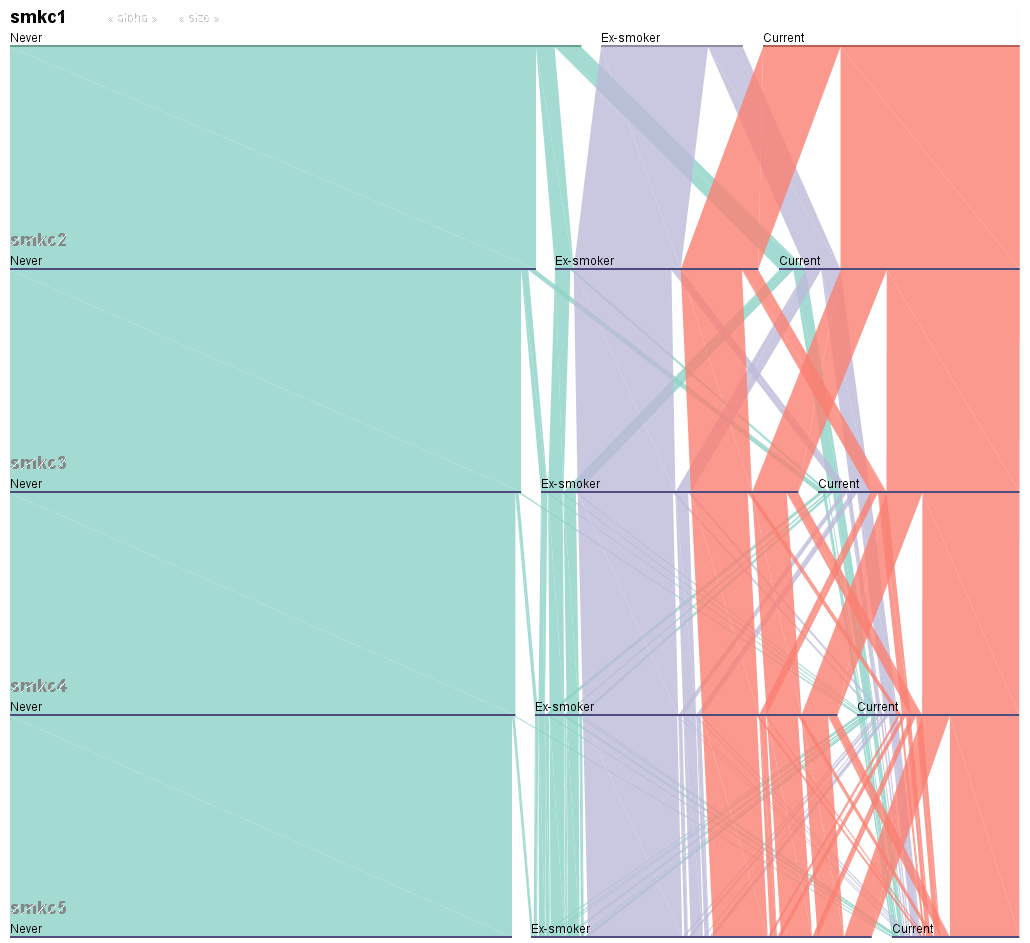

Supplement: Additional file 4: Figure S3 — Parallel sets diagram of smoking status transitions from survey waves 1 to 5. [file 1471-2288-14-32-S4.docx]
