# Supplementary material for: Visualising and modelling changes in categorical variables in longitudinal studies
Source: BMC Med Res Methodol. 2014 Feb 27;14:32. doi: 10.1186/1471-2288-14-32 (PMC3938907; doi:10.1186/1471-2288-14-32)

Figure S4: Plot and marginal distribution table of body mass index including a missing category over survey wave for the Australian Longitudinal Survey of Women’s Health


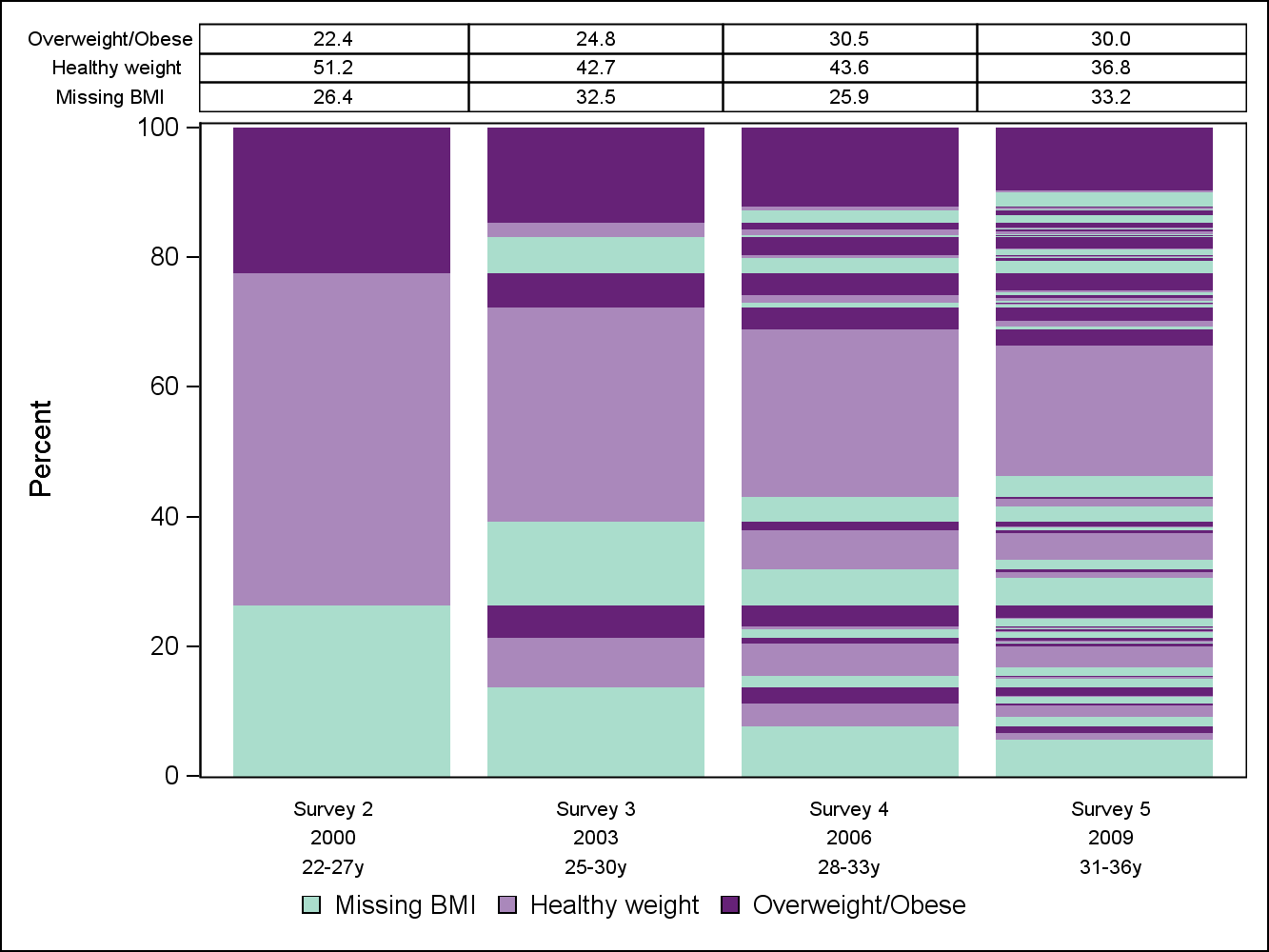

Supplement: Additional file 5: Figure S4 — Plot and marginal distribution table of body mass index group with a missing category over survey wave for the Australian Longitudinal Survey of Women’s Health. [file 1471-2288-14-32-S5.docx]
