# Supplementary material for: Visualising and modelling changes in categorical variables in longitudinal studies
Source: BMC Med Res Methodol. 2014 Feb 27;14:32. doi: 10.1186/1471-2288-14-32 (PMC3938907; doi:10.1186/1471-2288-14-32)

Figure S5: Plot and marginal distribution table of smoking status over survey wave for ex-smokers at survey wave 2


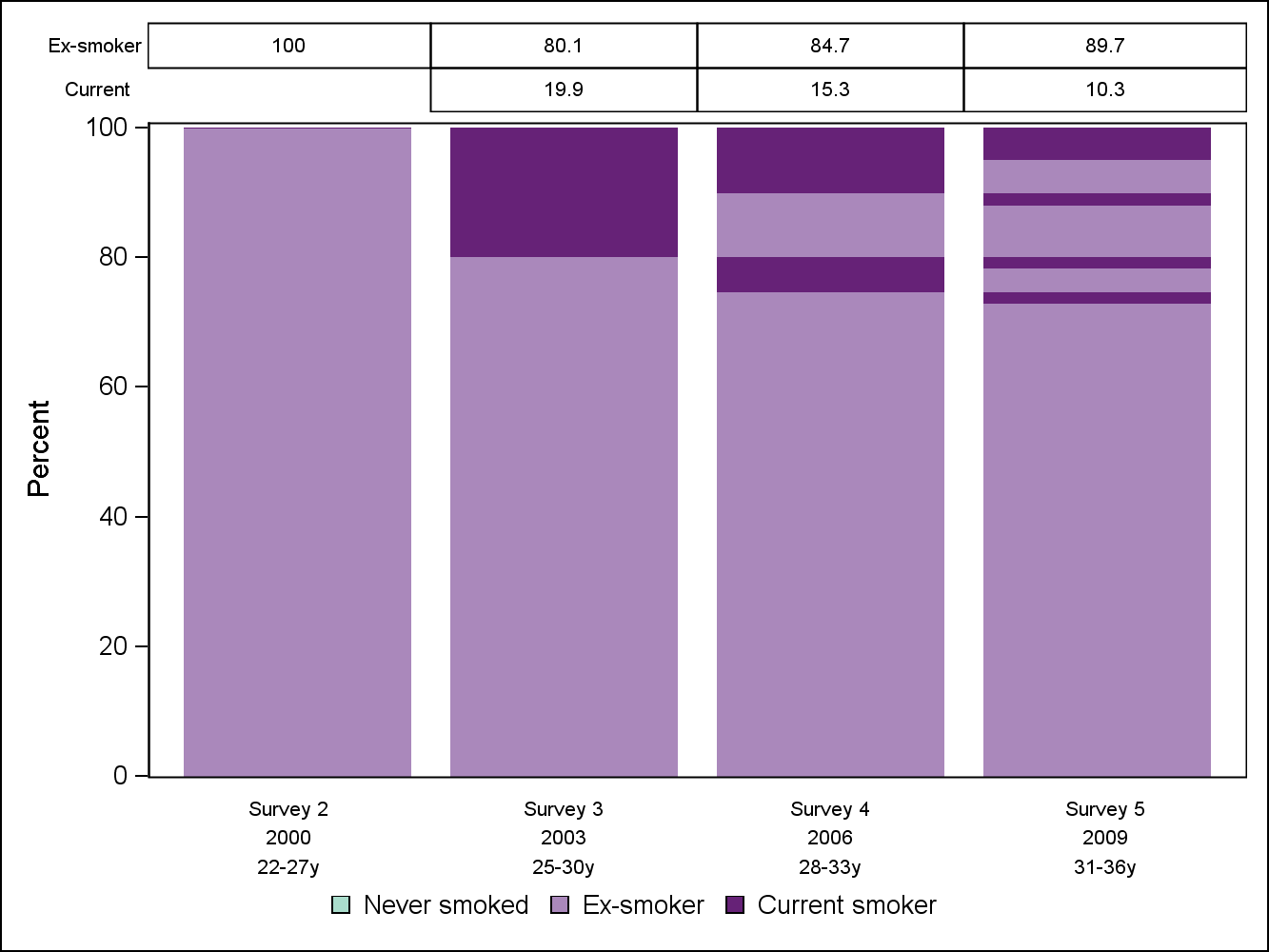

Supplement: Additional file 6: Figure S5 — Plot and marginal distribution table of smoking status over survey wave for ex-smokers at survey wave 2. [file 1471-2288-14-32-S6.docx]
